# Interferon-λs: Front-Line Guardians of Immunity and Homeostasis in the Respiratory Tract

**DOI:** 10.3389/fimmu.2017.01232

**Published:** 2017-09-29

**Authors:** Evangelos Andreakos, Maria Salagianni, Ioanna E. Galani, Ourania Koltsida

**Affiliations:** ^1^Laboratory of Immunobiology, Center for Clinical, Experimental Surgery and Translational Research, Biomedical Research Foundation of the Academy of Athens, Athens, Greece

**Keywords:** interferons, respiratory tract diseases, infection, asthma, cytokines, innate immunity

## Abstract

Type III interferons (IFNs), also termed lambda IFNs (IFNλs) or interleukins-28/29, constitute a new addition to the IFN family. They are induced upon infection and are particularly abundant at barrier surfaces, such as the respiratory and gastrointestinal tracts. Although they signal through a unique heterodimeric receptor complex comprising IFNLR1 and IL10RB, they activate a downstream signaling pathway remarkably similar to that of type I IFNs and share many functions with them. Yet, they also have important differences which are only now starting to unfold. Here, we review the current literature implicating type III IFNs in the regulation of immunity and homeostasis in the respiratory tract. We survey the common and unique characteristics of type III IFNs in terms of expression patterns, cellular targets, and biological activities and discuss their emerging role in first line defenses against respiratory viral infections. We further explore their immune modulatory functions and their involvement in the regulation of inflammatory responses during chronic respiratory diseases, such as asthma and chronic obstructive pulmonary disease. Type III IFNs are, therefore, arising as front-line guardians of immune defenses in the respiratory tract, fine tuning inflammation, and as potential novel therapeutics for the treatment of diverse respiratory diseases, including influenza virus infection and asthma.

## Introduction

Interferons (IFNs) have a long history. Type I IFNs were first discovered in 1957 as factors that “interfere” with viral replication (1). Type II IFN was identified a few years later, in 1965, as a molecule secreted by activated lymphocytes in response to antigenic stimulation (2). Yet, it was not until 2003 that a third type of IFNs also capable of “interfering” with viral infection termed type III IFNs, lambda IFNs (IFNλs) or interleukins-28/29 was described (3, 4). This raised new questions as to why nature needs three IFN systems and new challenges as to which specific roles each type of IFN fulfils.

Type III IFNs comprise four members in humans, IFNλ1/IL-29, IFNλ2/IL-28A, IFNλ3/IL-28B, IFNλ4, and two (IFNλ2/IL-28A, IFNλ3/IL-28B) in mice (3–5). By comparison, type I IFNs in humans and most mammals are encoded by about thirteen different IFNα genes, several more distantly related genes and pseudogenes, and a single IFNβ gene (6), while type II IFNs consist of only one gene, IFNγ (7). Type III IFNs signal through a unique heterodimeric receptor complex comprising IFNLR1 (IFNLRA), conferring ligand specificity, and IL10RB (IL-10R2), also shared with IL-10 family members and required for signaling. Type I IFNs signal through IFNAR1/IFNAR2 and IFNγ though IFNGR1/IFNGR2. Notably, all IFNs share the unique ability to activate large sets of genes, collectively known as interferon-stimulated genes (ISGs) that inhibit viral replication, degrade viral nucleic acids, and induce viral resistance to neighboring cells (8). As many ISGs are known to inhibit bacterial and parasitic infection as well (9, 10), this places IFNs at the center stage of antimicrobial immunity in mammals.

Among the various IFNs, type I IFNs have long been considered to constitute the primary antiviral and antibacterial defense mechanism in the body as they can be produced by almost any cell type upon infection and can signal to almost any cell type to confer protection (11). In contrast, IFNγ does not share this ubiquitous pattern of expression. Rather, its expression is restricted to NK cells and T cells, engaged later on during the antimicrobial immune response following the production of type I IFNs, IL-12, and other innate inflammatory cues, and involved in strengthening type I IFN-mediated defenses and regulating adaptive immunity (7). However, the discovery of type III IFNs that exhibit analogous activities and expression patterns with type I IFNs has complicated this paradigm, leading to the suggestion that type III IFNs may be more important in first line defenses at barrier surfaces such as the respiratory, gastrointestinal, and urogenital tracts (12–14). Here, we review the current literature implicating type III IFNs, referred throughout as IFNλs, in the regulation of immunity and homeostasis in the respiratory tract. We highlight unique antiviral and immune modulatory functions of IFNλs not shared with type I IFNs, and discuss why two apparently similar IFN systems are needed for optimal host protection.

## IFNλs Expression Patterns and Functions, and Comparison to Type I IFNs

IFNλs are induced in response to diverse pathogens including DNA and RNA viruses (3, 4, 15) as well as intracellular and extracellular bacteria (16, 17). In the respiratory tract, these comprise influenza viruses, rhinoviruses, respiratory syncytial viruses, *S. pneumonia, H. influenza, S. aureus*, and *M. tuberculosis*, all of which trigger high levels of IFNλs. Multiple pattern recognition receptors (PPRs) are involved in this process including endosomal toll-like receptors (TLR), such as TLR3, TLR7/8, and TLR9, and cytosolic sensors, such as RIG-I and MDA-5, recognizing double-stranded or single-stranded RNA, unmenthylated DNA, and other microbial structures (18).

Pattern recognition receptors are abundant in the respiratory epithelium and immune cells lying beneath the epithelial layer, sampling the airway lumen or residing in the lung parenchyma such as conventional and plasmacytoid dendritic cells (DCs), alveolar and interstitial macrophages, and monocytes. Interestingly, although these cells broadly respond to PRR engagement, expression of IFNλs is selective to specific cell types, most prominently epithelial cells and DCs (19–22), suggesting the involvement of additional epigenetic, transcriptional, and posttranscriptional regulation, which determines the ability of cells to make IFNλs. Indeed, RIG-I-like receptor signaling *via* mitochrondrial antiviral signaling protein (16) in peroxisomes or presence of transcriptional repressors, such as ZEB1 and BLIMP-1 (23), may provide such signals controlling IFNλ expression.

A surprising observation since the early days of their discovery was the ability of IFNλs to activate a remarkably similar downstream signaling cascade to that of type I IFNs. Despite the utilization of distinct receptor complexes, both IFNλs and type I IFNs trigger the JAK/STAT pathway, leading to the phosphorylation and nuclear translocation of STATs, the activation of interferon-regulatory factors, and the formation of the transcription complex IFN-stimulated gene factor 3 which is critically involved in the induction of ISGs (24, 25). Even on direct side-by-side comparisons in cultured cells, it has been difficult to distinguish type I from type III IFN responses (26–28). It has, therefore, been proposed that these cytokines share their antiviral activity (28–30), and indeed in numerous *in vitro* and *in vivo* studies IFNλ was shown to be as effective as type I IFNs in treating viral or bacterial infections (13, 14).

In an effort to explain why the organism employs two functional IFN systems with similar activities to confront infection, the idea of “ligand availability” was proposed (25). This was based on the notion that each unique infection induces a specific set of IFNs which accordingly determine the response. Although important, this “ligand-centric” view did not fit with many situations where both type I and type III IFNs are induced. The concept of “compartmentalization” was, therefore, put forward. This suggested that type III IFNs may be more important at barrier surfaces, such as the gastrointestinal epithelial layer, while type I IFNs may predominate once barrier surfaces are breached at the underlying tissues and the circulation. In support of that, IFNLR1 exhibits a very restricted pattern of expression compared to type I IFN receptors whose presence is ubiquitous, and is primarily found at epithelial origin cells although some leukocytes such as neutrophils can also express them (20, 21, 31, 32). Evidence for “compartmentalization” has come from recent work with intestinal pathogens indicating that IFNλs suffice to clear murine rotavirus, reovirus, or norovirus infection at the intestinal epithelium while type I IFNs are more important for preventing viral spread to the lamina propria and/or systemic dissemination (33–36). Still, compartmentalization alone may not suffice to explain the utility of two IFN systems. One report, in particular, has suggested a dispensable role for both type I and type III IFNs in murine rotavirus infection in the gastrointestinal tract, and only a temporal requirement of type III IFNs for protection against simian rotavirus infection (37). Moreover, in the respiratory track such clear-cut compartmentalization does not exist. Rather, it appears that IFNλs and type I IFNs exhibit distinct functions and activities that are only now starting to emerge.

## IFNλs Functions in Antiviral Immunity in the Respiratory Tract

The respiratory tract is among the sites of the body where type III IFNs are most abundantly expressed. The primary target of respiratory pathogens, such as influenza viruses and rhinoviruses, is the nose and tracheal epithelium of the upper respiratory tract but the lower airway epithelium and lung parenchyma can also be reached. Accordingly, primary nose and airway epithelial cells, and bronchial and alveolar epithelial cell lines, can all express high levels of IFNλs following infection in culture (31, 38–40). However, smooth muscle cells, fibroblasts, and immune cells such as conventional and plasmacytoid DCs can also express IFNλs (20, 22, 41, 42), suggesting that when the epithelial barrier is breached, additional sources of IFNλ production exist.

Type I IFNs are also induced by respiratory pathogens (11, 43). Respiratory epithelial cells express IFNβ while IFNα subtypes are primarily produced by immune cells. Smooth muscle cells and fibroblasts can also make them (43). Numerous studies over the years have demonstrated the key importance of type I IFNs in providing antiviral protection against influenza and parainfluenza viruses, rhinoviruses, respiratory syncytial viruses, adenoviruses, and others. *Ifnar^−/−^* animals, in particular, have been shown to be particularly susceptible to such infections while recombinant type I IFN treatment has been shown to prevent infection (11, 44).

**Figure 1:**
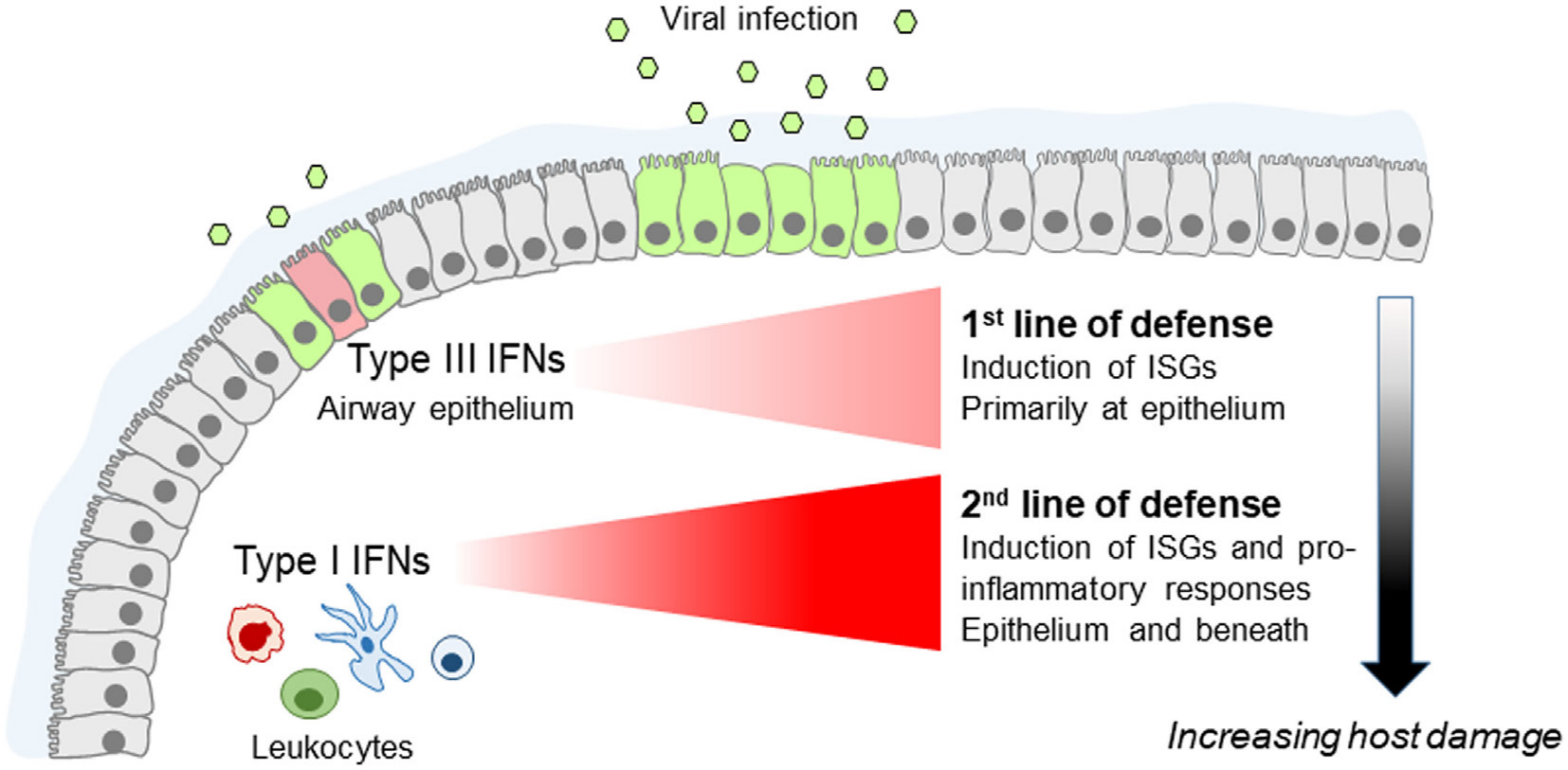
Fine tuning of the innate antiviral immune response by type I and type III interferons (IFNs) in the lung. Type III IFNs are produced first, upon infection of airway epithelial cells, and act as the first line of defense to limit virus spread at the epithelial barrier without triggering inflammation. If infection escapes type III IFN control, type I IFNs are induced that provide the second line of defense, enhancing viral resistance beyond the respiratory epithelium and activating pro-inflammatory responses essential for providing protection but also causing immunopathology.

IFNλs have, therefore, been considered to be of secondary importance till recently. Although initial studies in mice have shown that IFNλs are the predominant IFNs produced in response to infection (45) and that *Ifnlr1^−/−^Ifnar1^−/−^* animals are more susceptible to influenza virus infection compared to *Ifnar1^−/−^* animals, specific non-redundant functions of IFNλs in *Ifnlr1^−/−^* mice could not be described (20, 28, 46–48). IFNλs induce ISGs but so do type I IFNs. IFNλs can also activate NK cells when overexpressed (49), and endogenous IFNλ production seems to be required for optimal NK cell activity but these effects are indirect as NK cells do not express IFNLR1 (50). In addition, type I IFNs are direct and more potent activators of NK cells (51). Yet, recent more refined studies have started to uncover unique roles of IFNλs which cannot be substituted by type I IFNs. These have shown that IFNλs are the primary and earlier IFNs induced following viral infection, conferring viral resistance to the respiratory mucosa and limiting initial viral spread (32). When viral load is low, this suffices to confront infection. However, when viral load is high in the first place or escapes IFNλ control, type I IFNs are triggered in order to enhance the organism’s antiviral defenses. Accordingly, *Ifnlr1^−/−^* animals exhibit markedly enhanced viral burden following infection with low viral load and upregulated type I IFN levels, highlighting the essential role IFNλs play in these processes (Figure 1). Central to IFNλ-mediated antiviral protection is the respiratory epithelium. This is the site where IFNλs are first induced and primarily act, limiting initial viral spread. However, neutrophils are also important as they express high levels of IFNLR1 and respond to IFNλ signaling to deal with their uptaken viral load, preventing the virus from infecting neighboring epithelial cells (32).

Beyond the “timing” component, these studies have also uncovered a fundamental functional difference between type I and IFNλs. They demonstrated that although type I IFNs trigger robust pro-inflammatory responses characterized by the upregulation of diverse cytokines and chemokines, including TNF, IL-1b, and IL-6 (32, 52), IFNλs lack this function. They only induce the expression of ISGs without affecting the production of inflammatory mediators (32). Accordingly, recombinant IFNλ2 administration in experimental animals suppressed the immuno-inflammatory cascade triggered by respiratory viral infection, whereas IFNα exerted the opposite effect (32, 53). Interestingly, the expression of ISGs triggered by IFNλs follows slower and more prolonged kinetics compared to type I IFNs which induce faster but only transient expression of ISGs (26, 32, 54, 55). Central to the antiviral and/or pro-inflammatory activities of type I IFNs and IFNλs are neutrophils, which constitute the predominant leukocytes mediating initial antimicrobial immunity (56), and secreting cytokines and chemokines early during infection (57, 58). Although neutrophils respond to both IFNs to augment antiviral defenses, they exhibit pro-inflammatory activation only in response to type I IFNs (32), a finding that awaits confirmation in humans. Also, IFNλs directly affect neutrophil pro-inflammatory function, in both mice and humans, by suppressing reactive oxygen species production and degranulation of neutrophils, thereby limiting their tissue damaging functions and preserving barrier integrity (59).

Teleologically, this makes sense. Increased pro-inflammatory responses are needed for optimal protection against viral infection. However, they can also cause increased tissue damage, impaired respiratory function, and disease symptoms, and should not, therefore, be triggered unnecessarily. This is, in line with the emerging paradigm (schematically shown in Figure 1) placing type I IFNs as a second line of defense that only deal with respiratory infections that escape IFNλ control, at the expense though of host fitness.

## IFNλs Functions in Chronic Respiratory Diseases

**Figure 2:**
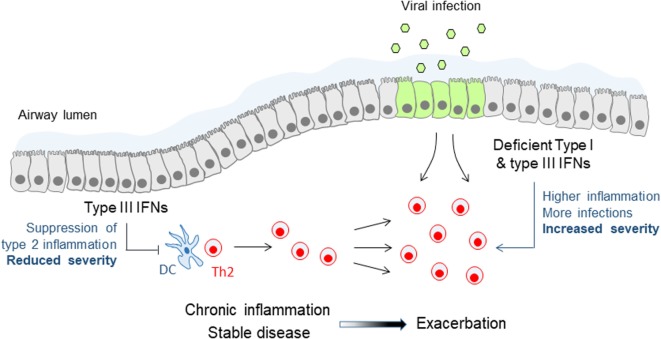
Immune modulatory and antiviral functions of type III interferons (IFNs) in asthma. Steady-state production of type III IFNs during stable asthma suppresses effector Th2 cell responses and keeps chronic inflammation and disease symptoms under control. Deficient or lower type III IFN production leads to reduced control of Th2 cell responses and chronic inflammation, and renders patients more susceptible to viral infections, both leading to more frequent and more severe asthma exacerbations. A similar mechanism of deficient type III IFN production may also account for chronic obstructive pulmonary disease exacerbations.

Research on IFNλs has mostly focused on their role in infections as these constitute the primary triggers of their expression *in vitro* and *in vivo*. Yet, it has been demonstrated that in settings of chronic inflammation IFNλs can also be induced independently of infectious insults, possibly through the action of cytokines and other inflammatory or environmental cues. Thus, during the development of allergic airway inflammation in mice significant levels of IFNλs have been detected in the bronchoalveolar lavage of these animals and have been shown to be required for reducing the inflammatory burden in the lung and keep allergic airway disease (AAD) under control (60). Accordingly, *Ifnlr1^−/−^* mice exhibit markedly worsened AAD while wild-type animals treated intranasally with recombinant IFNλ2 demonstrate significantly reduced type 2 inflammation and ameliorated disease. Although the molecular details of the mechanisms involved remain incompletely understood, these involve IFNλ signaling on lung conventional DCs, suppression of Th2 response, and induction of IFNγ (60). Interestingly, increased IFNλ mRNA levels have been detected in the sputum of asthmatic patients compared to healthy individuals, in the absence of evidence of viral infection, and have been shown to correlate in steroid-naïve patients with milder asthma symptoms, suggesting that IFNλs may also exhibit similar protective activities in human disease as well (61). Steady-state production of IFNλs appears, therefore, to be the key to keeping inflammation in asthma under control and reducing disease symptoms (Figure 2).

The effect of IFNλs to Th2 responses is not limited to the setting of AAD but may be of wider importance. IFNλs can suppress the development of primary immune responses *in vivo* as well (60). Also, IFNλs can inhibit Th2 responses *in vitro* in human cells through the reduction of GATA3 and IL-13, and possibly through the increase of IFNγ (62, 63). What remains to be clarified though is how exactly IFNλs are mediating these effects. There is a consensus that T cells do not directly respond to IFNλs to induce ISGs, the signature tag of type III IFN signaling (20, 59, 60). On the contrary, conventional DCs (60, 64, 65) and plasmacytoid DCs (20, 66–68) of either human or mouse origin, have been shown in several studies to upregulate ISGs and alter their function upon IFNλ stimulation. However, even in this case the situation is not crystal clear as there have also been reports that conventional (20, 59, 68) and plasmacytoid DCs (59) do not respond to IFNλs, possibly reflecting differences in their origin (e.g., spleen vs bone marrow or blood), culture or differentiation protocol, and cytokine environment (e.g., presence of IL-3, IL-4, GM-CSF, or other). More comprehensive studies addressing the responsiveness of various DC populations and subpopulations to IFNλ are, therefore, urgently needed. Noteworthy, it has been shown that IFNλs can induce the proliferation of Foxp3^+^ regulatory T cells *in vitro* (64, 65) but confirmation of these findings *in vivo* is still awaited.

IFNλs are also particularly important during asthma exacerbations. The induction of type I and type III IFNs following viral infection is deficient in allergic asthmatic patients with poorly controlled asthma, either because of the strongly Th2-polarized environment at the respiratory mucosa and the use of corticosteroids that generically suppress IFN production and function (e.g., through the induction of SOCS1) or because of epigenetic changes that prevent optimal IFNλ gene expression and translation (31, 69, 70). In either case, this renders allergic asthmatic patients distinctly susceptible to viral exacerbations of asthma, the main cause of hospitalizations and life-threatening situations in this disease (71). These exacerbations are characterized by sudden upregulation of epithelial-derived cytokines, such as IL-25 and IL-33, and rapid aggravation of type 2 responses in the airways, which can all be regulated by type I and type III IFNs (Figure 2). Indeed, a Phase II clinical study, administering inhalable IFNβ in a range of asthmatic patients with moderate to severe asthma, demonstrated significant improvement in the “difficult to treat” group of patients, highlighting the potential benefit of this approach (72). Although the treatment was overall well tolerated, the long-known adverse effects of type I IFNs, such as fever, diarrhea, and flu-like disease, are still an issue of concern. IFNλs are, therefore, currently being considered as a better alternative to type I IFNs for treating asthma exacerbations as they exhibit reduced adverse effects and a safer pharmacological profile.

Deficient IFN production of the respiratory epithelium has also been observed in chronic obstructive pulmonary disease (COPD), another disease characterized by frequent virally induced exacerbations. Bronchial epithelial cells from COPD patients are not capable of mounting a full IFN response upon viral infection (73). This is possibly due to cigarette smoke exposure as bronchial epithelial cells from smokers had significantly reduced IFNβ and IFNλ levels compared to non-smokers (74). Administration of recombinant IFNλs may, therefore, be beneficial for the treatment of COPD exacerbations as well. Whether IFNλs are also important at “steady state” during stable disease and whether they can be involved in other chronic respiratory diseases remains to be investigated.

## Conclusion and Future Directions

Over the last years, major progress in our understanding of the unique functions of IFNλs, not shared with type I IFNs, has taken place. This has revealed the importance of IFNλs in front-line antiviral defenses in the body, especially the respiratory and gastrointestinal tracts, acting in synergy with type I IFNs to fine tune immunity for optimal protection and minimal host damage. This has also uncovered the significance of IFNλs in keeping inflammation under control and preventing exacerbations in asthma, supporting their potential use for the treatment of diverse respiratory diseases. Despite that, key gaps of knowledge exist. Thus, it remains largely unexplored whether IFNλs are also important in immunity against bacterial or fungal infections of the respiratory tract, or barrier surfaces in general and how these are positioned by comparison to type I IFNs. It also remains unclear whether IFNλs are important in adaptive immune responses against infections, such as antibody and cytotoxic T cell responses, including immunological memory, which are well known to be affected by type I IFNs. Moreover, it remains to be established whether IFNλs are important in other chronic respiratory disorders beyond asthma and COPD, and how they can affect the course of the disease process. Further studies toward these directions are, therefore, urgently needed before these highly promising therapeutic candidates can be effectively exploited in the clinic.

## Author Contributions

EA, MS, IG, and OK have contributed to the writing of the manuscript. EA and OK have designed the graphs.

## Conflict of Interest Statement

The authors declare that the research was conducted in the absence of any commercial or financial relationships that could be construed as a potential conflict of interest.

## References

[1] Isaacs A Lindenmann J Virus interference. I. The interferon. Proc R Soc Lond B Biol Sci (1957) 147(927):258–67. 10.1098/rspb.1957.0048 13465720

[2] Wheelock EF Interferon-like virus-inhibitor induced in human leukocytes by phytohemagglutinin. Science (1965) 149(3681):310–1. 10.1126/science.149.3681.310 17838106

[3] Kotenko SV Gallagher G Baurin VV Lewis-Antes A Shen M Shah NK IFN-lambdas mediate antiviral protection through a distinct class II cytokine receptor complex. Nat Immunol (2003) 4(1):69–77. 10.1038/ni875 12483210

[4] Sheppard P Kindsvogel W Xu W Henderson K Schlutsmeyer S Whitmore TE IL-28, IL-29 and their class II cytokine receptor IL-28R. Nat Immunol (2003) 4(1):63–8. 10.1038/ni873 12469119

[5] Prokunina-Olsson L Muchmore B Tang W Pfeiffer RM Park H Dickensheets H A variant upstream of IFNL3 (IL28B) creating a new interferon gene IFNL4 is associated with impaired clearance of hepatitis C virus. Nat Genet (2013) 45(2):164–71. 10.1038/ng.2521 23291588 PMC3793390

[6] Pestka S Krause CD Walter MR. Interferons, interferon-like cytokines, and their receptors. Immunol Rev (2004) 202:8–32. 10.1111/j.0105-2896.2004.00204.x 15546383

[7] Schroder K Hertzog PJ Ravasi T Hume DA. Interferon-gamma: an overview of signals, mechanisms and functions. J Leukoc Biol (2004) 75(2):163–89. 10.1189/jlb.0603252 14525967

[8] Schneider WM Chevillotte MD Rice CM. Interferon-stimulated genes: a complex web of host defenses. Annu Rev Immunol (2014) 32:513–45. 10.1146/annurev-immunol-032713-120231 24555472 PMC4313732

[9] Boxx GM Cheng G. The roles of type I interferon in bacterial infection. Cell Host Microbe (2016) 19(6):760–9. 10.1016/j.chom.2016.05.016 27281568 PMC5847370

[10] Liehl P Zuzarte-Luis V Chan J Zillinger T Baptista F Carapau D Host-cell sensors for *Plasmodium* activate innate immunity against liver-stage infection. Nat Med (2014) 20(1):47–53. 10.1038/nm.3424 24362933 PMC4096771

[11] McNab F Mayer-Barber K Sher A Wack A O’Garra A Type I interferons in infectious disease. Nat Rev Immunol (2015) 15(2):87–103. 10.1038/nri3787 25614319 PMC7162685

[12] Galani IE Koltsida O Andreakos E. Type III interferons (IFNs): emerging master regulators of immunity. Adv Exp Med Biol (2015) 850:1–15. 10.1007/978-3-319-15774-0_1 26324342

[13] Wack A Terczynska-Dyla E Hartmann R. Guarding the frontiers: the biology of type III interferons. Nat Immunol (2015) 16(8):802–9. 10.1038/ni.3212 26194286 PMC7096991

[14] Lazear HM Nice TJ Diamond MS Interferon-lambda: immune functions at barrier surfaces and beyond. Immunity (2015) 43(1):15–28. 10.1016/j.immuni.2015.07.001 26200010 PMC4527169

[15] Ank N West H Bartholdy C Eriksson K Thomsen AR Paludan SR Lambda interferon (IFN-lambda), a type III IFN, is induced by viruses and IFNs and displays potent antiviral activity against select virus infections *in vivo*. J Virol (2006) 80(9):4501–9. 10.1128/JVI.80.9.4501-4509.2006 16611910 PMC1472004

[16] Odendall C Dixit E Stavru F Bierne H Franz KM Durbin AF Diverse intracellular pathogens activate type III interferon expression from peroxisomes. Nat Immunol (2014) 15(8):717–26. 10.1038/ni.2915 24952503 PMC4106986

[17] Bierne H Travier L Mahlakoiv T Tailleux L Subtil A Lebreton A Activation of type III interferon genes by pathogenic bacteria in infected epithelial cells and mouse placenta. PLoS One (2012) 7(6):e39080. 10.1371/journal.pone.0039080 22720036 PMC3375250

[18] Iwasaki A Pillai PS Innate immunity to influenza virus infection. Nat Rev Immunol (2014) 14(5):315–28. 10.1038/nri3665 24762827 PMC4104278

[19] Spann KM Tran KC Chi B Rabin RL Collins PL Suppression of the induction of alpha, beta, and lambda interferons by the NS1 and NS2 proteins of human respiratory syncytial virus in human epithelial cells and macrophages [corrected]. J Virol (2004) 78(8):4363–9. 10.1128/JVI.78.8.4363-4369.2004 15047850 PMC374276

[20] Ank N Iversen MB Bartholdy C Staeheli P Hartmann R Jensen UB An important role for type III interferon (IFN-lambda/IL-28) in TLR-induced antiviral activity. J Immunol (2008) 180(4):2474–85. 10.4049/jimmunol.180.4.2474 18250457

[21] Sommereyns C Paul S Staeheli P Michiels T. IFN-lambda (IFN-lambda) is expressed in a tissue-dependent fashion and primarily acts on epithelial cells *in vivo*. PLoS Pathog (2008) 4(3):e1000017. 10.1371/journal.ppat.1000017 18369468 PMC2265414

[22] Coccia EM Severa M Giacomini E Monneron D Julkunen I Cella M Viral infection and toll-like receptor agonists induce a differential expression of type I and lambda interferons in human plasmacytoid and monocyte-derived dendritic cells. Eur J Immunol (2004) 34(3):796–805. 10.1002/eji.200324610 14991609

[23] Siegel R Eskdale J Gallagher G Regulation of IFN-lambda1 promoter activity (IFN-lambda1/IL-29) in human airway epithelial cells. J Immunol (2011) 187(11):5636–44. 10.4049/jimmunol.1003988 22058416

[24] Kotenko SV IFN-lambdas. Curr Opin Immunol (2011) 23(5):583–90. 10.1016/j.coi.2011.07.007 21840693 PMC3196341

[25] Durbin RK Kotenko SV Durbin JE. Interferon induction and function at the mucosal surface. Immunol Rev (2013) 255(1):25–39. 10.1111/imr.12101 23947345 PMC5972370

[26] Bolen CR Ding S Robek MD Kleinstein SH. Dynamic expression profiling of type I and type III interferon-stimulated hepatocytes reveals a stable hierarchy of gene expression. Hepatology (2014) 59(4):1262–72. 10.1002/hep.26657 23929627 PMC3938553

[27] Kohli A Zhang X Yang J Russell RS Donnelly RP Sheikh F Distinct and overlapping genomic profiles and antiviral effects of interferon-lambda and -alpha on HCV-infected and non-infected hepatoma cells. J Viral Hepat (2012) 19(12):843–53. 10.1111/j.1365-2893.2012.01610.x 23121362 PMC3511888

[28] Crotta S Davidson S Mahlakoiv T Desmet CJ Buckwalter MR Albert ML Type I and type III interferons drive redundant amplification loops to induce a transcriptional signature in influenza-infected airway epithelia. PLoS Pathog (2013) 9(11):e1003773. 10.1371/journal.ppat.1003773 24278020 PMC3836735

[29] Zhou Z Hamming OJ Ank N Paludan SR Nielsen AL Hartmann R Type III interferon (IFN) induces a type I IFN-like response in a restricted subset of cells through signaling pathways involving both the Jak-STAT pathway and the mitogen-activated protein kinases. J Virol (2007) 81(14):7749–58. 10.1128/JVI.02438-06 17507495 PMC1933366

[30] Doyle SE Schreckhise H Khuu-Duong K Henderson K Rosler R Storey H Interleukin-29 uses a type 1 interferon-like program to promote antiviral responses in human hepatocytes. Hepatology (2006) 44(4):896–906. 10.1002/hep.21312 17006906

[31] Contoli M Message SD Laza-Stanca V Edwards MR Wark PA Bartlett NW Role of deficient type III interferon-lambda production in asthma exacerbations. Nat Med (2006) 12(9):1023–6. 10.1038/nm1462 16906156

[32] Galani IE Triantafyllia V Eleminiadou EE Koltsida O Stavropoulos A Manioudaki M Interferon-lambda mediates non-redundant front-line antiviral protection against influenza virus infection without compromising host fitness. Immunity (2017) 46(5):875–890e6. 10.1016/j.immuni.2017.04.025 28514692

[33] Pott J Mahlakoiv T Mordstein M Duerr CU Michiels T Stockinger S IFN-lambda determines the intestinal epithelial antiviral host defense. Proc Natl Acad Sci U S A (2011) 108(19):7944–9. 10.1073/pnas.1100552108 21518880 PMC3093475

[34] Hernandez PP Yang I Schwierzeck V Nguyen N Guendel F Gronke K Interferon-lambda and interleukin 22 act synergistically for the induction of interferon-stimulated genes and control of rotavirus infection. Nat Immunol (2015) 16(7):698–707. 10.1038/ni.3180 26006013 PMC4589158

[35] Mahlakoiv T Hernandez P Gronke K Diefenbach A Staeheli P Leukocyte-derived IFN-alpha/beta and epithelial IFN-lambda constitute a compartmentalized mucosal defense system that restricts enteric virus infections. PLoS Pathog (2015) 11(4):e1004782 10.1371/journal.ppat.1004782 25849543 PMC4388470

[36] Nice TJ Baldridge MT McCune BT Norman JM Lazear HM Artyomov M Interferon-lambda cures persistent murine norovirus infection in the absence of adaptive immunity. Science (2015) 347(6219):269–73. 10.1126/science.1258100 25431489 PMC4398891

[37] Lin JD Feng N Sen A Balan M Tseng HC McElrath C Distinct roles of type I and type III interferons in intestinal immunity to homologous and heterologous rotavirus infections. PLoS Pathog (2016) 12(4):e1005600 10.1371/journal.ppat.1005600 27128797 PMC4851417

[38] Okabayashi T Kojima T Masaki T Yokota S Imaizumi T Tsutsumi H Type-III interferon, not type-I, is the predominant interferon induced by respiratory viruses in nasal epithelial cells. Virus Res (2011) 160(1–2):360–6. 10.1016/j.virusres.2011.07.011 21816185

[39] Spann KM Baturcam E Schagen J Jones C Straub CP Preston FM Viral and host factors determine innate immune responses in airway epithelial cells from children with wheeze and atopy. Thorax (2014) 69(10):918–25. 10.1136/thoraxjnl-2013-204908 24811725 PMC4174127

[40] Wang J Oberley-Deegan R Wang S Nikrad M Funk CJ Hartshorn KL Differentiated human alveolar type II cells secrete antiviral IL-29 (IFN-lambda 1) in response to influenza A infection. J Immunol (2009) 182(3):1296–304. 10.4049/jimmunol.182.3.1296 19155475 PMC4041086

[41] Yang K Puel A Zhang S Eidenschenk C Ku CL Casrouge A Human TLR-7-, -8-, and -9-mediated induction of IFN-alpha/beta and -lambda is IRAK-4 dependent and redundant for protective immunity to viruses. Immunity (2005) 23(5):465–78. 10.1016/j.immuni.2005.09.016 16286015 PMC7111074

[42] Lauterbach H Bathke B Gilles S Traidl-Hoffmann C Luber CA Fejer G Mouse CD8alpha+ DCs and human BDCA3 + DCs are major producers of IFN-lambda in response to poly IC. J Exp Med (2010) 207(12):2703–17. 10.1084/jem.20092720 20975040 PMC2989774

[43] Trinchieri G Type I interferon: friend or foe? J Exp Med (2010) 207(10):2053–63. 10.1084/jem.20101664 20837696 PMC2947062

[44] Wang BX Fish EN The Yin and Yang of viruses and interferons. Trends Immunol (2012) 33(4):190–7. 10.1016/j.it.2012.01.004 22321608 PMC7106503

[45] Jewell NA Cline T Mertz SE Smirnov SV Flano E Schindler C Lambda interferon is the predominant interferon induced by influenza A virus infection *in vivo*. J Virol (2010) 84(21):11515–22. 10.1128/JVI.01703-09 20739515 PMC2953143

[46] Mordstein M Kochs G Dumoutier L Renauld JC Paludan SR Klucher K Interferon-lambda contributes to innate immunity of mice against influenza A virus but not against hepatotropic viruses. PLoS Pathog (2008) 4(9):e1000151 10.1371/journal.ppat.1000151 18787692 PMC2522277

[47] Mordstein M Neugebauer E Ditt V Jessen B Rieger T Falcone V Lambda interferon renders epithelial cells of the respiratory and gastrointestinal tracts resistant to viral infections. J Virol (2010) 84(11):5670–7. 10.1128/JVI.00272-10 20335250 PMC2876583

[48] Mahlakoiv T Ritz D Mordstein M DeDiego ML Enjuanes L Muller MA Combined action of type I and type III interferon restricts initial replication of severe acute respiratory syndrome coronavirus in the lung but fails to inhibit systemic virus spread. J Gen Virol (2012) 93(Pt 12):2601–5. 10.1099/vir.0.046284-0 22956738

[49] Wang Y Li T Chen Y Wei H Sun R Tian Z. Involvement of NK cells in IL-28B-mediated immunity against influenza virus infection. J Immunol (2017) 199(3):1012–20. 10.4049/jimmunol.1601430 28637903

[50] Souza-Fonseca-Guimaraes F Young A Mittal D Martinet L Bruedigam C Takeda K NK cells require IL-28R for optimal *in vivo* activity. Proc Natl Acad Sci U S A (2015) 112(18):E2376–84. 10.1073/pnas.1424241112 25901316 PMC4426428

[51] Paolini R Bernardini G Molfetta R Santoni A. NK cells and interferons. Cytokine Growth Factor Rev (2015) 26(2):113–20. 10.1016/j.cytogfr.2014.11.003 25443799

[52] Davidson S Crotta S McCabe TM Wack A Pathogenic potential of interferon alphabeta in acute influenza infection. Nat Commun (2014) 5:3864 10.1038/ncomms4864 24844667 PMC4033792

[53] Davidson S McCabe TM Crotta S Gad HH Hessel EM Beinke S IFNlambda is a potent anti-influenza therapeutic without the inflammatory side effects of IFNalpha treatment. EMBO Mol Med (2016) 8(9):1099–112. 10.15252/emmm.201606413 27520969 PMC5009813

[54] Maher SG Sheikh F Scarzello AJ Romero-Weaver AL Baker DP Donnelly RP IFNalpha and IFNlambda differ in their antiproliferative effects and duration of JAK/STAT signaling activity. Cancer Biol Ther (2008) 7(7):1109–15. 10.4161/cbt.7.7.6192 18698163 PMC2435218

[55] Jilg N Lin W Hong J Schaefer EA Wolski D Meixong J Kinetic differences in the induction of interferon stimulated genes by interferon-alpha and interleukin 28B are altered by infection with hepatitis C virus. Hepatology (2014) 59(4):1250–61. 10.1002/hep.26653 23913866 PMC3909557

[56] Galani IE Andreakos E. Neutrophils in viral infections: current concepts and caveats. J Leukoc Biol (2015) 98(4):557–64. 10.1189/jlb.4VMR1114-555R 26160849

[57] Mantovani A Cassatella MA Costantini C Jaillon S. Neutrophils in the activation and regulation of innate and adaptive immunity. Nat Rev Immunol (2011) 11(8):519–31. 10.1038/nri3024 21785456

[58] Tecchio C Micheletti A Cassatella MA. Neutrophil-derived cytokines: facts beyond expression. Front Immunol (2014) 5:508. 10.3389/fimmu.2014.00508 25374568 PMC4204637

[59] Broggi A Tan Y Granucci F Zanoni I IFN-lambda suppresses intestinal inflammation by non-translational regulation of neutrophil function. Nat Immunol (2017) 18:1084–93. 10.1038/ni.3821 28846084 PMC5701513

[60] Koltsida O Hausding M Stavropoulos A Koch S Tzelepis G Ubel C IL-28A (IFN-lambda2) modulates lung DC function to promote Th1 immune skewing and suppress allergic airway disease. EMBO Mol Med (2011) 3(6):348–61. 10.1002/emmm.201100142 21538995 PMC3377081

[61] Bullens DM Decraene A Meyts I De Boeck K Dupont LJ Ceuppens JL. Type III IFN-lambda mRNA expression in sputum of adult and school-aged asthmatics. Clin Exp Allergy (2008) 38(9):1459–67. 10.1111/j.1365-2222.2008.03045.x 18564328

[62] Jordan WJ Eskdale J Srinivas S Pekarek V Kelner D Rodia M Human interferon lambda-1 (IFN-lambda1/IL-29) modulates the Th1/Th2 response. Genes Immun (2007) 8(3):254–61. 10.1038/sj.gene.6364382 17361203

[63] Dai J Megjugorac NJ Gallagher GE Yu RY Gallagher G. IFN-lambda1 (IL-29) inhibits GATA3 expression and suppresses Th2 responses in human naive and memory T cells. Blood (2009) 113(23):5829–38. 10.1182/blood-2008-09-179507 19346497

[64] Mennechet FJ Uze G. Interferon-lambda-treated dendritic cells specifically induce proliferation of FOXP3-expressing suppressor T cells. Blood (2006) 107(11):4417–23. 10.1182/blood-2005-10-4129 16478884

[65] Dolganiuc A Kodys K Marshall C Saha B Zhang S Bala S Type III interferons, IL-28 and IL-29, are increased in chronic HCV infection and induce myeloid dendritic cell-mediated FoxP3+ regulatory T cells. PLoS One (2012) 7(10):e44915 10.1371/journal.pone.0044915 23071503 PMC3468613

[66] Megjugorac NJ Gallagher GE Gallagher G. Modulation of human plasmacytoid DC function by IFN-lambda1 (IL-29). J Leukoc Biol (2009) 86(6):1359–63. 10.1189/jlb.0509347 19759281

[67] Yin Z Dai J Deng J Sheikh F Natalia M Shih T Type III IFNs are produced by and stimulate human plasmacytoid dendritic cells. J Immunol (2012) 189(6):2735–45. 10.4049/jimmunol.1102038 22891284 PMC3579503

[68] Zhang S Kodys K Li K Szabo G Human type 2 myeloid dendritic cells produce interferon-lambda and amplify interferon-alpha in response to hepatitis C virus infection. Gastroenterology (2013) 144(2):414–425e7. 10.1053/j.gastro.2012.10.034 23089201 PMC3568254

[69] Edwards MR Regamey N Vareille M Kieninger E Gupta A Shoemark A Impaired innate interferon induction in severe therapy resistant atopic asthmatic children. Mucosal Immunol (2013) 6(4):797–806. 10.1038/mi.2012.118 23212197 PMC3684776

[70] Wark PA Johnston SL Bucchieri F Powell R Puddicombe S Laza-Stanca V Asthmatic bronchial epithelial cells have a deficient innate immune response to infection with rhinovirus. J Exp Med (2005) 201(6):937–47. 10.1084/jem.20041901 15781584 PMC2213100

[71] Jackson DJ Sykes A Mallia P Johnston SL. Asthma exacerbations: origin, effect, and prevention. J Allergy Clin Immunol (2011) 128(6):1165–74. 10.1016/j.jaci.2011.10.024 22133317 PMC7172902

[72] Djukanovic R Harrison T Johnston SL Gabbay F Wark P Thomson NC The effect of inhaled IFN-beta on worsening of asthma symptoms caused by viral infections. A randomized trial. Am J Respir Crit Care Med (2014) 190(2):145–54. 10.1164/rccm.201312-2235OC 24937476 PMC4226052

[73] Mallia P Message SD Gielen V Contoli M Gray K Kebadze T Experimental rhinovirus infection as a human model of chronic obstructive pulmonary disease exacerbation. Am J Respir Crit Care Med (2011) 183(6):734–42. 10.1164/rccm.201006-0833OC 20889904 PMC3081284

[74] Wu W Zhang W More S Booth JL Duggan ES Liu L Cigarette smoke attenuates the RIG-I-initiated innate antiviral response to influenza infection in two murine models. Am J Physiol Lung Cell Mol Physiol (2014) 307(11):L848–58. 10.1152/ajplung.00158.2014 25260755 PMC4254961

